# Obtaining Aromatic Extracts from Portuguese *Thymus mastichina* L. by Hydrodistillation and Supercritical Fluid Extraction with CO_2_ as Potential Flavouring Additives for Food Applications

**DOI:** 10.3390/molecules27030694

**Published:** 2022-01-21

**Authors:** Júlia C. Kessler, Vanessa A. Vieira, Isabel M. Martins, Yaidelin A. Manrique, Andreia Afonso, Patrícia Ferreira, Filipa Mandim, Isabel C. F. R. Ferreira, Lillian Barros, Alírio E. Rodrigues, Madalena M. Dias

**Affiliations:** 1 Laboratory of Separation and Reaction Engineering-Laboratory of Catalysis and Materials (LSRE-LCM), Faculty of Engineering, University of Porto, 4200-465 Porto, Portugal; juliakessler@fe.up.pt (J.C.K.); vanessa.vieira@deifil.pt (V.A.V.); yjam@fe.up.pt (Y.A.M.); arodrig@fe.up.pt (A.E.R.); dias@fe.up.pt (M.M.D.); 2 Associate Laboratory in Chemical Engineering (ALiCE), Faculty of Engineering, University of Porto, 4200-465 Porto, Portugal; 3 Centro de Investigação de Montanha (Mountain Research Center) (CIMO), Polytechnic Institute of Bragança, Campus de Santa Apolónia, 5300-253 Bragança, Portugal; filipamandim@ipb.pt (F.M.); iferreira@ipb.pt (I.C.F.R.F.); lillian@ipb.pt (L.B.); 4 DEIFIL-Deifil Technology, Serzedelo, 4839-704 Póvoa de Lanhoso, Portugal; andreia.afonso@deifil.pt (A.A.); patricia.ferreira@deifil.pt (P.F.)

**Keywords:** SFE-CO_2_, natural odours, food ingredients, thymol rich extracts, terpenoids

## Abstract

Humans often respond to sensory impulses provided by aromas, and current trends have generated interest in natural sources of fragrances rather than the commonly used synthetic additives. For the first time, the resulting aroma of a selected culture of *Thymus mastichina* L. was studied as a potential food ingredient. In this context, dried (DR) and fresh (FR) samples were submitted to carbon dioxide (CO_2_) supercritical extraction (SFE) and hydrodistillation (HD) methods. The extracts were characterised according to their volatile composition by GC-MS, cytotoxicity against a non-tumour cell culture, and sensory attributes (odour threshold and olfactive descriptors). The most abundant aromas were quantified, and the analysis performed by GC-MS revealed an abundance of terpenoids such as thymol chemotype, followed by the precursors α-terpinene and *p*-cymene. DR and FR extracts (EX) obtained from SFE-CO_2_ show the highest content of thymol, achieving 52.7% and 72.5% of the isolated volatile fraction. The DR essential oil (EO) contained the highest amount of terpenoids, but it was also the most cytotoxic extract. In contrast, SFE-CO_2_ products showed the lowest cytotoxic potential. Regarding FR-OE, it had the lowest extraction yield and composition in aroma volatiles. Additionally, all samples were described as having green, fresh and floral sensory notes, with no significant statistical differences regarding the odour detection threshold (ODT) values. Finally, FR-EX of *T. mastichina* obtained by SFE-CO_2_ presented the most promising results regarding food application.

## 1. Introduction

Lipophilic volatile compounds play a notable influence on the olfactory marketing strategy in the food, cosmetic and fragrance industries. Selective oils from natural origins are recognised as one of the main approaches to product improvement, whether to extend shelf life, in alternative medicine treatments or for sensory purposes [1,2]. The compounds extracted from plants, namely essential oils, have distinct chemical compositions related not only to the species itself but also to agriculture practices, soil properties, climate conditions and stage of development [3]. Over the last century, advances in the knowledge of chemical routes have proven the contribution of the environmental state in the secondary metabolism of plants [4].

Chemically unstable and easily isomerized, volatile fractions obtained by conventional extraction procedures (e.g., HD, steam distillation (SD), maceration (MAC), and Soxhlet (SOX)) show significant chemical alterations. The presence of light, heat and the existence of an organic solvent are some of the conditions that promote the decomposition of unsaturated or esterified molecules [5]. The thermal hydrolysis effect was observed in HD products by the formation of the *p*-cymene molecule, an aromatic monoterpene and precursor of the thymol and carvacrol (both isomers) compounds [6,7]. This reaction can occur rapidly through dehydrogenated and oxygenated γ-terpinene, respectively [1,8]. However, in the last three decades, SFE has increased the naturalness of the odour of the extracted products [9]. SFE is a green extraction technology and typically uses CO_2_ as a solvent at a critical pressure of 73.8 bar and a minimum temperature of 32.1 °C [5]. In addition to its safety, chemical stability, low cost and availability characteristics [10], CO_2_ can change its solvent capability by changing pressure and temperature, thus affecting the density of the fluid and the extraction efficiency [7,11]. Generally recognised as safe by the Food and Drug Administration [9], CO_2_ as a supercritical fluid is highly selective and favours the extraction of thermolabile bioactive compounds [7].

As shown by Costa et al. [12], by using a SFE-CO_2_ process with gradual decompression, different compounds can be collected in order of their molecular weight in different separators. In this work, the first separator was designed to capture the heavier substances (mainly sesquiterpenoids such as β-caryophyllene), and the second separator was designed for lighter components such as monoterpenoids (e.g., γ-terpinene, linalool and camphor). Depending on the system pressure, no EX was recovered in both separators simultaneously, as shown by García-Risco et al. [13] using pressures from 150 to 400 bar and a temperature of 40 °C. No EX was obtained in the first separator at low pressure. Nevertheless, the product of the second separator has a similar chemical composition to those obtained in the severest extraction conditions, differing just in their relative concentration and yield.

Comparable results were shown for several raw materials [7,12,13,14,15,16,17]. Thereby, to guarantee the selectivity and high solubility of the volatile fraction, normally the indicated SFE-CO_2_ conditions are around 80 to 100 bar and 40 to 50 °C [11,16]. Moreover, studies have reported a reduction of selectivity of the supercritical products when higher pressure and temperature values were applied, resulting in the co-extraction of long-chain lipids compounds [17].

Aiming to present these well-known concepts for traditional plant material from Northern Portugal, the main genotypes and sensory attributes of *T. mastichina* were studied. *T. mastichina* is an endemic and undershrub Iberian Peninsula herb, reaching more than 50 cm in height and blossoming from April to June [18]. Between the 215 species of genus *Thymus* [3], Portuguese *T. mastichina* shows a few explorative works, especially in the early 2000’s [18,19,20,21]. The most embracing study so far was done by Salgueiro et al. [22] in 1997, giving significant advances in data information of the selected plant. Delgado et al. [23] studied *T. mastichina* collected from 20 different populations in Spain.

Regarding the numerous studies found in the literature about *Thymus* spp. aromatic plants, the chemical composition of its main molecules is listed in Table 1, where the species and origin of production, extraction procedures, respective conditions, yields and prevailing genotypes are also listed.

Thyme’s dual activities highlight the herb for edible applications. First, the plant is widely used as a natural additive due to its expressive antimicrobial property against common food pathogens (e.g., *Staphylococcus aureus*) [23]. On the other hand, the terpenoid compounds (e.g., thymol, carvacrol, *p*-cymene) show desirable improvements in foods as flavouring agents [1]. Its pleasant aromatic properties play an interesting role in replacing synthetic ingredients generally introduced in industrial processes [9]. In fact, through the organoleptic characteristics of food products, the deepest human sensations and feelings can be revealed. Thus, exploring these perceptions constitute a potential strategy for marketing purposes.

This work compares the performance of DR and FR *T. mastichina* extracts obtained from different extraction methods, namely SFE-CO_2_ and HD. Descriptive and quantitative sensory assays (QDA) determine the fragrances attributes and odour detection threshold of samples and, finally, the cytotoxicity potential is defined within a safe concentration range for the addition of extracted oils in food products.

To the authors’ best knowledge, this is the first time that the *T. mastichina* extracts from SFE-CO_2_ and the HY fraction obtained in the HD extraction have been studied. Finally, this work aims to present a proposal for the use of *T. mastichina* EX as a compound rich in odours and which can be used as a flavouring additive for food products.

## 2. Materials and Methods

### 2.1. Chemicals

Carbon dioxide (CAS 124-38-9, food industry-grade, 99.9%) was supplied from Linde (Lisbon, Portugal). Thymol (CAS 89-83-8, 98%), eucalyptol (CAS 470-82-6, 99%) and camphor (CAS 76-22-2, 96%) were acquired from Alfa Aesar (Madrid, Spain). α-pinene (CAS 80-56-8, 98%), thyme oil (CAS 8007-46-3, FCC, FG) and alkane C8–C40 (ref. 40147-U) standard solution were purchased from Sigma Aldrich (Madrid, Spain). Verbenone (CAS 1196-01-6, 99%) and *n*-hexane (CAS 110-54-3, 99%) were obtained from Supelco (Madrid, Spain).

### 2.2. Plant Material

*T. mastichina* herb was cultivated under controlled conditions at Deifil Technology (Póvoa de Lanhoso, Braga, Portugal). After the development of micropropagated seedlings from the same mother plant, the aerial material of the plant was collected in May 2020 and subjected to an air-drying process at 40 °C (Venticell, MMM Medcenter, Planegg, Germany) until constant weight, achieving 82.06 ± 1.14% (*w*/*w*) of water content.

### 2.3. Essential Oil and Hydrolate Isolation

The HD procedure was conducted with 75 g of FR and DR raw plant in duplicate and independent assays. The extraction was carried out using a conventional extraction apparatus. In this methodology, the essential oils of *Thymus* are evaporated by heating a mixture of water and plant materials followed by the liquefaction of the vapours in a condenser [25]. The extraction was carried out for 3 h with deionized water, as suggested by the European Pharmacopeia [26]. The experimental apparatus also comprises a condenser and a decanter to collect the condensate and to separate essential oils from water. Both products, essential oil and hydrolate, obtained in each extraction experiment were properly separated by their density differences. The HY fraction was kept under ultra-fast freezing as a preparation stage for the lyophilization process (55C, CoolSafe, Beverwijk, The Netherland) under vacuum at −50 °C for 72 h. All solid fractions obtained were kept under −20 °C, while the oil fraction was stored at 4 °C until further analysis.

### 2.4. SFE-CO_2_ T. mastichina L. Extraction

The supercritical extractor developed by Gomes et al. [27] was used for the SFE-CO_2_ process, and following this work, the volatile compounds were obtained under fixed conditions of pressure and temperature, namely 80 bar and 50 °C. A stainless-steel extraction cell capable of containing 1 L of CO_2_ and designed to perform experiments in static mode was inserted into the system with 30 g of *T. mastichina* plant using FR and DR samples. To guarantee full recovery of the extracted content after 2 h, a CO_2_ re-alimentation cycle was performed. The products were collected through the regulation valves (VR1 operated at 40 bar and VR2 at 6 bar) inside 2 separators (S1 and S2). The recovered EX was stored at 4 °C for further characterization.

The extraction yields for both extraction methods was obtained by

(1) $$Yield\;\left(\%\right)=\left[\frac{m_{\mathrm{essential}\;\mathrm{oil}}\left(g\right)}{m_{\mathrm{sample}\;(\mathrm{DW})}\left(g\right)}\right]\times 100$$

### 2.5. Volatile Composition Determined by GC-MS Assay

The volatile compounds of *T. mastichina* products were analysed by gas chromatography (GC) with an ion-trap mass spectrometer (MS) (TQ8040 NX Triple Quadrupole, Shimadzu, Japan) using a cross-bonded fused column (30 m × 0.25 mm, 0.25 µm film thickness) for low-polarity phases (Rxi-5Sil MS, Restek, Bellefonte, USA). The GC-MS instrument operated with a splitless injector and automatic sampler (AOC-20s+i). All samples were diluted in *n*-hexane (GC grade), and a volume of 1 µL was injected at 290 °C, carried at 1 mL·min^−1^ by ultrapure He.

The isothermal oven program was initially set at 40 °C for 1 min, then raised to 200 °C for 2 min at a rate of 7 °C·min^−1^, followed by an increase to 250 °C for 2 min at 15 °C·min^−1^ and finally to 280 °C for 1 min at 20 °C·min^−1^. The mass scanning range was kept at *m*/*z* 40–500, while the ion and interface temperature were at 250 °C and 260 °C, respectively.

The resulting aroma fractions are presented in relative concentration (%), obtained by the GC peak areas for each identified compound. Molecules were discriminated based on the National Institute of Standards & Technology (NIST 21, 27, 107, 147, Gaithersburg, MD, USA) mass spectra. To confirm the identification, the retention time of the homologous series of alkanes (C8–C40), analysed under the same chromatographic conditions, was used to calculate the linear retention indices (LRI) by the Kovats retention index equation [28]. The comparison between the obtained LRI and those reported in the literature guarantees the precision of the development chromatographic method and allows for the quantification of the selected molecules according to the calibration curves of the respective analytical standards.

### 2.6. Odour Perception and Description of T. mastichina L. Volatile Compounds

Olfactory evaluations are affected by vapour pressure, relative composition in the gas and liquid phases, atmospheric pressure and temperature conditions, in addition to the psychophysics background [9].

In accordance with the recommendations of the International Standard ISO 8586:2012 [29], a panel of 12 judges was trained to discriminate the most promising samples of *T. mastichina* on the perception of minimum odour concentration, ODT, and index their respective sensory notes by Quantitative Descriptive Analysis (QDA). Subsequently, the commercial standard was incorporated from this study phase to evaluate the achieved products.

To carry out the experiments, a clean, ventilated and well-lighted room was used with a controlled temperature of 20 °C. This room was also free from annoying noises. Furthermore, in the face of the SARS-CoV-2 crises, social and hygienic precautions against the virus were also adopted.

#### 2.6.1. Odour Detection Threshold (ODT)

Odour analysis was performed for the individual products following the instructions of ISO 13301:2018 [30]. Thus, samples in a concentration range of 1.0 × 10^−5^ to 1.0 × 10^−2^ µg·mL^−1^ covered with cotton and conditioned in polypropylene flasks were presented to each panellist. The odour provided by the solutions was properly inhaled, and the minimal odour perception was identified.

#### 2.6.2. Quantitative Descriptive Analysis (QDA)

Aqueous samples with a concentration range of 1.0 × 10^3^ µg·mL^−1^ were all evaluated individually by the panel members. The descriptors used in the sensory analysis were carefully selected in the literature according to the main compounds of *T. mastichina*. An in-depth discussion indicated woody, green, fresh, citrus, floral, sweet, spicy, fruity and oily as the appropriate odour notes. The methodology was based on ISO 11035:1994 [31], and panellists scored the odour notes on an unstructured scale between 0 “not very intense” and 9 “very intense” to record their perceptions.

### 2.7. Cytotoxicity Analysis

All aqueous and lipophilic-based products were subjected to a cytotoxic assay using a monkey non-tumour cell line, Vero (kidney cells, ATCC CCL81.4, Gaithersburg, MD, USA). Kidneys play an important role in vertebrate organisms due to their osmoregulatory and excretion functions. Therefore, as the envisioned application is for food purposes, the potential cytotoxic effect of the extracts was evaluated through these cells. The samples were dissolved in aqueous DMSO (50%, *v*/*v*) at a final concentration of 8 mg∙mL^−1^ and further diluted in the range of 400 to 6.25 µg∙mL^−1^. Following the protocol previously described by Barros et al. [32], the sulforhodamine B assay was performed, where ellipticine was used as a positive control, while the negative control was represented by a suspension of cells. The tests were performed independently (three independent assays) and in triplicate. The obtained results were expressed in GI50 values (the concentration that inhibited 50% of cell proliferation).

### 2.8. Statistical Analysis

The extraction yields, sensory ODT values and cytotoxicity analysis were carried out using ANOVA and Tukey tests (α = 0.05) performed in Statistica StatSoft (version 64, Tulsa, OK, USA).

## 3. Results and Discussion

### 3.1. Extraction Yields of T. mastichina L. Product

**Figure 1:**
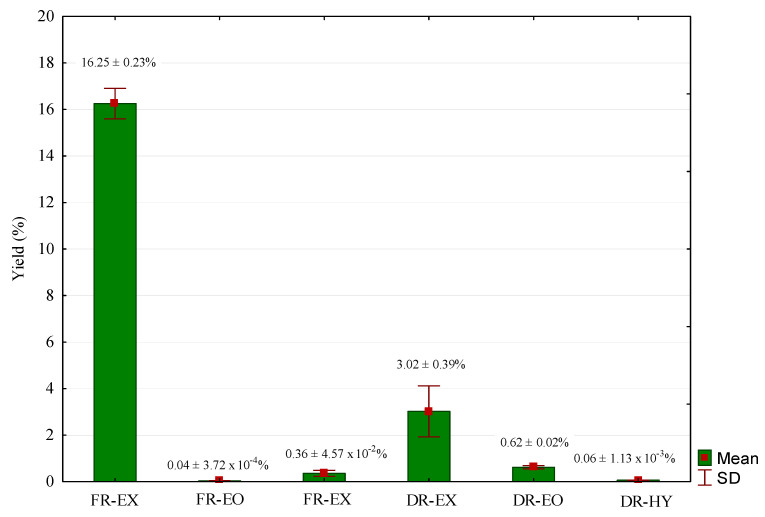
Extraction yields of DR and FR *T. mastichina* fractions obtained by SFE-CO_2_ and HD method, presented in DW.

Significant differences were observed in the values of extraction yields between the two methods (SFE-CO_2_ and HD) and between types of samples (FR or DR) (Figure 1). The SFE-CO_2_ assays resulted in a mix of extractable compounds, collected only in the S1 separator. FR-EX obtained from SFE-CO_2_ showed the highest extraction yield (16.25 ± 0.23%), followed by DR-EX with 3.02 ± 0.39%, determined in DW (Dry Weight). These results are an effect of the high water content in the raw material [33], which reached 82.06 ± 1.14% (*w*/*w*); this value was considered in the extraction yield calculations. Additionally, the differences can be explained by three possible phenomena: (i) the dissolution of water in the supercritical solvent increased the solubility of the *Thymus* compounds; (ii) the hydration of the samples decreased the mass transfer resistance by the diffusion mechanism of intracellular compounds to the particle surface of the *Thymus*; and (iii) there were significant losses of matter during the drying process [34]. Statistically, all HD products presented similar extraction yield values, ranging from 0.04 ± 0.0004% (EO-FR) to 0.62 ± 0.02% (EO-DR). Previous works report EO of *T. mastichina* of 1.3% [19], 2.3% [20] and 1.4 to 3.5% [18], far from the percentages achieved in the present study. Regarding the HY obtained for FR and DR plant samples, traces of solid fraction were obtained after the lyophilization process: 196.45 mg and 222.30 mg in FR- and DR-HY, corresponding to 0.36 ± 0.05% and 0.06 ± 0.001%, respectively.

### 3.2. Chemical Composition of T. mastichina L. Product

The chemical variation of the *Thymus* family goes beyond the extraction method and offers a remarkable diversification according to the species and origin of growth [3]. However, as expected, the SFE-CO_2_ method showed the highest selectivity in the extracted compounds in relation to the EO-HD results. The EO had a large source of different molecules, with a total of 39; this is as compared to 25 of the FR-EX from SFE-CO_2_. Similar results were obtained in the DR raw material, with 30 molecules for the EO in comparison to 22 compounds from the DR-EX obtained by SFE-CO_2_. Usually, HD extractions show a lower selectivity than SFE-CO_2_. For example, Costa et al. [12] identified 44 and 20 compounds in *T. lotocephalus* extracts obtained by HD and SFE-CO_2_ methods, respectively.

**Figure 2:**
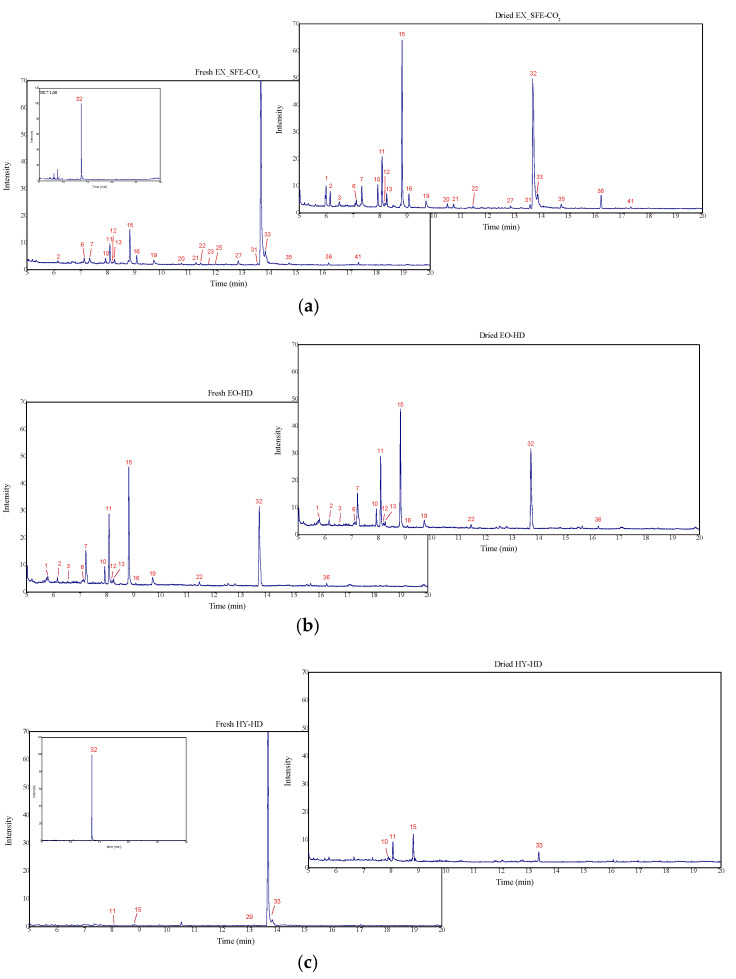
Chromatographic profile of FR and DR *T. mastichina* products analysed in GC-MS and identified as (**a**) EX obtained by SFE-CO_2_, (**b**) EO extracted by HD and (**c**) HY provided from the HD method. The samples were analysed at different injection concentrations: (**a**) 2.5 g/L, (**b**) 0.01 g/L and (**c**) HY 0.1 g/L, respectively. The numbers at each peak represent the compounds identified in Table 2.

Table 2 and Figure 2 present the chemical composition of *T. mastichina* samples obtained in this work, and Table 3 presents the mass quantification of the main molecules. Thymol is the main molecule found in SFE-CO_2_-EX, representing 72.4% and 52.7% or 1511.3 ± 602.3 μg∙g^−1^ and 282.1 ± 7.5.7 μg∙g^−1^ of FR and DR samples, respectively. In relation to the lyophilized HY, a relative composition of 98.6% to FR-HY and 11.5% to DR-HY was obtained. The high relative concentration of thymol was previously reported for *T. vulgaris* and *T. zygis* HY, 77.1% and 61.9%, respectively [35].

The precursors γ-terpinene and *p*-cymene also showed significant values, mainly in the EO samples. The FR sample was composed of 30.6% of γ-terpinene and 10.3% of *p*-cymene, while the DR-EO presented 35.5% and 17.6%, respectively. In contrast, the representativeness of both molecules was lower in SFE-CO_2_-EX, reaching 13.6% (FR-EX) and 28.8% (DR-EX). Following the chemical route, the relative influence of thymol increases as the contribution of γ-terpinene and *p*-cymene decreases. Tohidi et al. [8] observed a positive correlation between the formation molecules, mentioning that *p*-cymene does not present itself as a final product.

As previously mentioned, the most abundant molecules present in the obtained extracts were quantified, and the results are shown in Table 3. The richest extract was the DR-EO, revealing 2565.5 ± 208.7 μg∙g^−1^ of thymol, 609.4 ± 28.3 μg∙g^−1^ of γ-terpinene and 337.0 ± 20.3 μg∙g^−1^ of *p*-cymene molecules. In total, the sample presented an amount of 3700.7 μg∙g^−1^. As observed through the yield results, FR-HY had a higher total mass concentration (2429.1 μg∙g^−1^) than the FR-EO fraction (524.8 μg∙g^−1^), which suggests a considerable amount of the oil fraction solubilized in hydrophilic fraction. On the other hand, the HY of the DR plant was composed only of 4.3 μg∙g^−1^. Furthermore, the methodology to concentrate the organic products in HY is limited in terms of energy and time costs, although it is easy and cheap to produce them [35].

Different volatile compounds were previously referenced in Portuguese *T. mastichina* herbs. Eucalyptol, linalool and camphor stand out in EO obtained by the HD method [18,19,21,22]. β-pinene and limonene were also described as main molecules in samples cultivated in the North and South regions [19,20]. Although the present sample contains these compounds in smaller proportion, the chemotype corresponds to *T. vulgaris* plants collected in Algeria [44], Italy [45], Portugal [11], Romania [46] and Spain [15,24], or even to *T. zygis* species from Portugal [9].

The thymol chemotype is a natural phenolic monoterpene group synthesized by the transformation of γ-terpinene and *p*-cymene precursors. Both thymol and carvacrol isomers are indicated as the end of the biosynthetic chain catalysed by the two-step oxidation of γ-terpinene under the action of the cytochrome P450 enzyme (γ-terpinene synthase), which is able to modify the polarity of the compounds and make them more water-soluble [47]. Geranyl diphosphate (GPP) plays an important role in the initial chemical route, followed by the α-terpineol intermediate [48]. Through a series of steps, the molecules are converted into γ-terpinene, which, by cyclization and carbonisation reactions, form the *p*-cymene molecule [3]. *p*-cymene is a by-product of the premature release of substrate from the active site [8,47], and, by terpene synthase (TPS) activity, it produces thymol or carvacrol molecules when it reaches the last step of biosynthetic conversion. The TPS offers a wide range of ideal characteristics to modify the metabolic profile of terpenes [3]. At the same time, the final products are formed directly via γ-terpinene hydroxylation [47,48].

Moreover, α-thujene, β-myrcene, 3-carene and β-caryophyllene had some influence on the chemical profile of *T. mastichina* plant, mainly in DR samples. *S*-verbenone was only found in FR-EX (0.4% or 61.8 ± 21.1 μg∙g^−1^), while notable amounts of eucalyptol were measured in DR-OE (0.5% or 123.8 ± 9.7 μg∙g^−1^) compared to the other lipophilic fractions. The resulting mass composition demonstrates that even a low relative concentration can indicate the presence of a compound rich in the molecules of interest.

### 3.3. Cytotoxicity Assay of T. mastichina L. Products

The most promising products were selected to perform the successive odour and cytotoxicity characteristics. Table 4 presents the cytotoxicity and ODT results. Concerning the cytotoxicity potential, significant differences were reached between EX, EO and HY. However, the values did not show statistical differences between the hydration state of FR samples. For example, in SFE-CO_2_-EX, the GI50 values ranged from 235 ± 7 µg·mL^−1^ to 271 ± 2 µg·mL^−1^ in the FR and DR samples, respectively. Instead, the commercial EO was statistically equal in both EO obtained in this study, which reveals the average toxicological profile of 83.5 ± 0.1 µg·mL^−1^, while FR-EO and DR-EO reached 122 ± 12 µg·mL^−1^ and 65 ± 1 µg·mL^−1^, respectively. Nikolic et al. [49] showed higher cytotoxic activity in OE obtained from *T. serpyllum* (7.02 µg·mL^−1^ < GI50 < 52.69 µg·mL^−1^) and *T. algenensis* EO (62.12 µg·mL^−1^ < GI50 < 64.79 µg·mL^−1^). Furthermore, the results for *T. vulgaris* EO (76.02 µg·mL^−1^ < GI50 < 180.40 µg·mL^−1^) ranged around the FR-OE cytotoxic potential [49], and the HY fraction did not show toxicity potential (GI50 > 400 µg·mL^−1^).

### 3.4. Sensory Odour Evaluation of T. mastichina L. Products

The ODT results are expressed in Table 4, and according to the panellist’s sensitivity, all the evaluated values were similar with 95% of confidence. The standard EO was the most easily detected product at a concentration of 1.0 × 10^−4^ µg·mL^−1^, while the DR-EO had the highest concentration value (5.0 × 10^−3^ µg·mL^−1^). FR- and DR-EX showed a concentration of 6.3 × 10^−4^ µg·mL^−1^ and 3.0 × 10^−4^ µg·mL^−1^ as minimum values to reflect some sensory perception, respectively.

**Figure 3:**
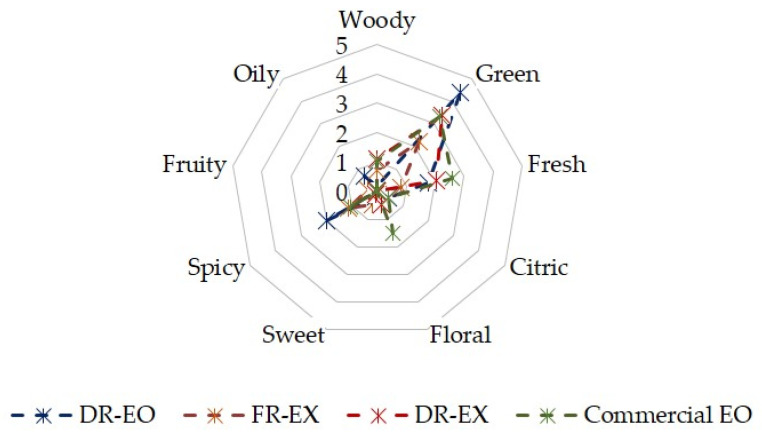
Sensory description and intensity of selected *T. mastichina* samples.

The human nose is a powerful tool to predict the dominant odours in a mixture [50], and, as already indicated above, the *T. mastichina* herb is comprised of volatile terpene compounds. Its influence on odour description was evaluated under controlled conditions to ensure the accuracy of human measurement, and the results were compared with the literature. The most pronounced attributes mentioned by the mean scores of 12 panellists were green, fresh and floral (Figure 3). The DR-EO was the only one with an oily note (0.7 points), with an emphasis on the green fragrance (4.4). The commercial EO has a strong green (3.3), fresh (2.6) and woody (1.0) description, like DR-EX. The FR-EX obtained by SFE-CO_2_ exhibited the smoothest analysis among all samples, presenting green (2.2), spicy (1.1) and fresh (0.8) aromas. The intensity results agreed with the respective ODT values.

EX of *T. vulgaris* plant were related with spicy, pungent, herbaceous and phenol-like odour characteristics. The product was obtained with supercritical CO_2_ under 167 bar and 40 °C. Changing the pressure to 104 bar, the descriptors’ intensity decreased, while the EO extracted by HD presented lower note values plus the burn attribute [7]. These findings suggest the thymol molecule as the key to the thyme aroma [7,51]. Furthermore, this distinct odour profile has been pointed out for three different subspecies of Algeria *Thymus*, ranging from a sweet herbaceous fragrance, phenolic herbaceous odour and fresh camphoraceous notes [52].

Moreover, using validated models with ODT results, relative odour intensities and resulting odour values (OV), Teixeira et al. [9] classified three *Thymus* families. According to the data, herbaceous nuances prevailed in the samples, in addition to citrus, woody, floral and phenolic sensory attributes.

In fact, the herbaceous term codifies the aromatic characteristics provided by most Lamiaceae species [7,51] and offers a subjective interpretation of the odour description. Therefore, although it does not represent a fingerprint of the *T. mastichina*, the description of the odour presented is akin to the profile of the compounds previously mentioned. Furthermore, the obtained products revealed a promising opportunity to enhance the flavouring trajectory of processed foods in a safe range of cytotoxicity, especially to the FR-EX.

Scanning all samples and features analysed in this work, FR-EX obtained by SFE-CO_2_ appears most promising. The sample reached a balance between the extraction yields, mass composition of the main compounds and toxicity value, and it has been rarely studied previously [21]. To the best of the authors’ knowledge, this is the first time that the fresh *T. mastichina* species has been sensorially described.

## 4. Conclusions

Terpinene-rich extracts were isolated from *T. mastichina* herb by means of green technologies, namely HD and SFE-CO_2_. The predominant thymol chemotype and its precursors, γ-terpinene and *p*-cymene, were the main molecules responsible for the sensory profile described as green, fresh and floral fragrance.

Additionally, no ODT results showed significant differences, and both SFE-CO_2_ products achieved the lowest toxicity among the lipophilic products. Moreover, the FR-HY fraction showed a higher selectivity of the compound, although with a low yield on a DW. The analysed characteristics of the extracted products suggested the FR-EX obtained through SFE-CO_2_ as the most promising sample to use as a flavouring additive for food applications.

According to the authors’ best knowledge, the species *T. mastichina* was studied for the first time under supercritical conditions as well as regarding its sensory profile. Hydrodistillation and supercritical fluid extraction were applied to the respective final extraction yields for fresh and dry samples of thymus plant material. The extracts’ chemical composition, sensorial attributes and cytotoxic potential were evaluated. Safe concentration ranges of the most promising *T. mastichina* products were defined by cytotoxicity measurements and ODT values, and the significant differences between the extraction yields, sensory ODT values and cytotoxicity analysis were achieved using statistical analysis, namely ANOVA and Tukey tests.

The resulting research presented an unexplored point of view for one of the most studied aromatic plants in recent decades and gives a suggestion for the use of *T. mastichina* extract as compound rich in odours and which can be used as a flavouring additive for food products.

## Figures and Tables

**Table 1:** Dataset of the main extraction results of *Thymus* species performed with HD and SFE-CO_2_ methods over the last ten years.

Species	Origin	Yields	Main Volatile Compounds		References
			HD	SFE-CO_2_	
*T. fontqueri*, *T. x. citriodorus* and *T. zygis* subsp. *gracilis*	Portugal	HD (0.67 to 1.26%) and SFE-CO_2_ (<0.05 to 0.77%)	*T. fontqueri*: carvacrol (60.3%), γ-terpinene (25.8%) and *p*-cymene (6.3%); *T. x. citriodorus*: geraniol (76.1%), geranial (5.3%) and neral (3.7%) and *T. zygis*: thymol (41.0%), *p*-cymene (16.0%), and γ-terpinene (10.0%)	*T. fontqueri*: *p*-cymene (41.0%), γ-terpinene (25.8%) and carvacrol (2.6%); *T. x. citriodorus*: geraniol (66.5%), geranial (8.6%) and thymol (7.6%) and *T. zygis*: thymol (33.7%), *p*-cymene (10.5%) and carvacrol (8.7%)	[9]
*T. munbyanus* subsp. *coloratus* and *T. munbyanus* subsp. *munyanus*	Algeria	HD (0.09 to 0.11%) and SFE-CO_2_ (0.4%)	Camphor (11.7%), geranyl acetate (6.3%) and β-terpinyl acetate (5.1%)	*E*-nerolidol (2.0 to 13.7%), 4-terpineol (0.2 to 10.6%) and camphor (1.1. to 7.6%)	[10]
*T. lotocephalus*	Portugal	HD (0.30%) and SFE-CO_2_ (2.24 to 7.76%)	Linalool (10.4%), camphor (8.0%) and caryophyllene oxide (8.1%)	Camphor (1.2 to 7.9%), borneol (6.1 to 7.5%) and *cis*-linalool oxide (0.2 to 7.2%)	[12]
*T. vulgaris* L.	Egypt	HD (1.00%) and SFE-CO_2_ (0.32 to 1.28%)	*p*-cymene (35.7%), thymol (33.2 %), and γ-terpinene (9.5%)	Thymol (45.2 to 82.6%), fenipentol (n.d. to 8.48%) and phytol isomer (n.d. to 7.2%)	[7]
	Portugal	~0.023 kg/kg	Thymol (41.6%), *p*-cymene (28.9%) and γ-terpinene (5.1%)	Thymol (36.3%), *p*-cymene (24.4%) and thymoquinone (6.2%)	[11]
	Spain	0.8 to 1.1%	Thymol (35.4 to 41.6%), *p*-cymene (28.9 to 34.8%) and γ-terpinene (5.1 to 7.0%)	Thymol (19.5 to 40.8%), *p*-cymene (10.0 to 42.6%) and γ-terpinene (0.8 to 6.9%)	[15]
	Spain	-	Thymol (35.4%), *p*-cymene (34.7%) and γ-terpinene (7.0%)	Thymol (36.8%), *p*-cymene (28.6%) and γ-terpinene (4.1%)	[24]

HD: hydrodistillation, SFE-CO_2_: dioxide carbon supercritical extraction, n.d.: not detected.

**Table 2:** Chemical profile of the extracted products obtained from *T. mastichina* herb, analysed in GC-MS and obtained by SFE-CO_2_ and HD methods.

No	Compound	LRI ^a^		FR						DR						Sensory Descriptors
			RT (min)	EX		EO		HY		EX		EO		HY		
**1**	α-Thujene	926	5988	tr	-	1.8	0.2	-	-	2.0	0.1	2.0	0.1	-	-	pine earthy, turpentine, fresh, sweet and woody [36,37]
**2**	α-Pinene	933	6140	0.6	0.1	0.6	0.0	-	-	1.5	0.0	1.1	0.0	-	-	herbaceous and green [9]
**3**	Camphene	949	6480	tr	-	tr	-	-	-	0.3	0.0	0.4	-	-	-	camphor, woody and herbal [36,37]
**4**	Sabinene	972	6963	tr	-	tr	-	-	-	-	-	tr	-	-	-	pine, turpentine, woody, terpenic, spicy and citrus [36,37]
**5**	β-Pinene	977	7067	-	-	tr	-	-	-	-	-	tr	-	-	-	green, piney, woody and green [37,38]
**6**	Amyl vinyl carbinol	980	7128	0.9	0.1	tr	-	-	-	0.5	0.0	0.3	0.0	-	-	mushroom, earthy, green and herbs [39,40,41,42]
**7**	β-Myrcene	988	7313	1.0	0.1	1.8	0.0	-	-	2.0	0.0	2.7	0.2	-	-	herbaceous, woody and floral [9]
**8**	3-octanol	997	7490	-	-	tr	-	-	-	-	-	tr	-	-	-	earthy, mushroom and herbal [37]
**9**	α-Phellandrene	1006	7681	-	-	tr	-	-	-	-	-	-	-	-	-	citrus, herbaceous and terpenic [37]
**10**	3-Carene	1016	7908	1.3	0.5	2.8	0.0	-	-	2.5	0.1	4.14	0.3	8.7	0.1	citrus and sweet [9,37]
**11**	*p*-Cymene	1023	8067	4.6	0.5	10.3	0.3	0.2	0.0	6.5	0.2	17.6	1.1	34.2	0.9	green, fresh, rubber, terpenic, woody and spicy [37,38]
**12**	d-Limonene	1029	8180	0.2	0.0	0.4	0.1	-	-	0.2	0.0	0.4	0.0	-	-	citrus, fresh and sweet [36,37]
**13**	Eucalyptol	1031	8239	1.1	0.2	0.9	0.1	-	-	1.2	0.0	0.5	0.0	-	-	woody, citrus, green, herbaceous and spicy [9,38,43]
**14**	β-*cis*-Ocimene	1047	8580	-	-	tr	-	-	-	-	-	tr	-	-	-	green and woody [9]
**15**	γ-Terpinene	1058	8809	9.1	0.4	30.6	0.7	0.3	0.1	22.4	0.8	35.5	1.7	45.6	0.7	herbaceous, woody, piney and fruity [37,38]
**16**	β-Terpineol	1070	9062	1.8	0.4	tr	-	-	-	1.5	0.8	0.2	0.0	-	-	woody, pungent and earthy [37]
**17**	Sabinene hydrate	1071	9090	-	-	tr	-	-	-	-	-	-	-	-	-	herbaceous, minty and oriental [9,37]
**18**	Terpinolene	1086	9414	-	-	tr	-	-	-	-	-	tr	-	-	-	herbaceous, woody and green [9]
**19**	β-Linalool	1099	9695	1.0	0.2	1.3	0.0	-	-	1.0	0.1	1.9	0.2	-	-	floral, lavender, citrus, woody and green [36,37,38]
**20**	Camphor	1147	10.719	0.4	0.2	-	-	-	-	0.4	0.1	-	-	-	-	camphoraceous and herbal [37]
**21**	Borneol	1172	11.263	0.4	0.1	tr	-	-	-	0.7	0.0	tr	-	-	-	herbaceous, oriental and woody [9]
**22**	4-Terpineol	1180	11.444	0.4	0.1	1.4	0.1	-	-	0.2	0.0	0.7	0.1	-	-	spicy, woody and oriental [9,37]
**23**	α-Terpineol	1194	11.743	0.1	0.0	tr	-	-	-	-	-	tr	-	-	-	terpenic, pine and woody [37]
**24**	*trans*-Dihydrocarvone	1201	11.900	-	-	tr	-	-	-	-	-	-	-	-	-	-
**25**	*S*-Verbenone	1206	11.993	0.4	0.1	-	-	-	-	-	-	-	-	-	-	camphoreous [37]
**26**	β-Citral	1240	12.691	-	-	tr	-	-	-	-	-	-	-	-	-	citrus [9,37]
**27**	Unknown	1247	12.827	0.8	0.3	-	-	-	-	0.3	0.1	-	-	-	-	-
**28**	Nerol	1249	12.873	-	-	tr	-	-	-	-	-	-	-	-	-	floral, citrus and green [9]
**29**	Unknown	1264	13.168	-	-	-	-	0.3	0.0	-	-	-	-	-	-	-
**30**	α-Citral	1277	13.440	-	-	tr	-	-	-	-	-	tr	-	-	-	citrus [9,37]
**31**	Bornyl acetate	1284	13.574	0.5	0.1	-	-	-	-	0.5	0.0	-	-	-	-	camphor, woody, pine, balsamic, herbal and spicy [37]
**32**	Thymol	1290	13.700	72.4	28.9	48.2	3.1	98.6	1.6	52.7	1.4	31.8	2.6	-	-	herbaceous [9,37]
**33**	Carvacrol	1298	13.857	1.0	0.6	tr	-	0.5	0.1	0.9	0.1	tr	-	11.5	1.3	herbaceous and woody [9]
**34**	Elixene	1334	14.546	-	-	tr	-	-	-	-	-	-	-	-	-	-
**35**	Thymol acetate	1344	14.750	0.4	0.0	tr	-	-	-	0.5	0.1	tr	-	-	-	thymol, sweet and balsamic [37]
**36**	β-Caryophyllene	1422	16.220	0.8	0.2	-	-	-	-	2.1	0.1	0.7	0.1	-	-	spicy, woody and sweet [9,37]
**37**	Copaene	1433	16.410	-	-	tr	-	-	-	-	-	-	-	-	-	woody, spicy and honey [37]
**38**	Citronellyl propionate	1438	16.505	-	-	tr	-	-	-	-	-	-	-	-	-	floral and green [37]
**39**	β-Farnesene	1454	16.785	-	-	tr	-	-	-	-	-	tr	-	-	-	woody, citrus and herbal [37]
**40**	α-Humulene	1458	16.872	-	-	tr	-	-	-	-	-	tr	-	-	-	woody [9,37]
**41**	Germacrene D	1483	17.317	0.8	0.1	tr	-	-	-	0.2	0.0	-	-	-	-	-
**42**	Germacrene B	1498	17.595	-	-	tr	-	-	-	-	-	-	-	-	-	woody, earthy and spicy [37]
**43**	Caryophylene oxide	1585	19.088	-	-	tr	-	-	-	-	-	-	-	-	-	woody and herbaceous [9]
**44**	γ-Eudesmol	1612	19.725	-	-	tr	-	-	-	-	-	tr	-	-	-	-
**45**	α-Cadinol	1630	20.292	-	-	tr	-	-	-	-	-		-	-	-	herbaceous and woody [9,37]
**46**	Isopropyl myristate	1723	22.850	-	-	-	-	-	-	-	-	tr	-	-	-	-
**47**	Isopropyl palmitate	1927	26.102	-	-	-	-	-	-	-	-	tr	-	-	-	-
	**Identified total**		**90.2**		**100.0**		**99.7**		**99.8**			**100.0**		**100.0**		

^a^ LRI: linear retention indices calculated through Kovat’s retention index equation for series of alkanes C8–C40 (ref. 40147-U) using a cross-bonded fused column (Rxi-5Sil MS, Restek, Bellefonte, PA, USA) in GC-MS; EX: extract obtained by carbon dioxide supercritical fluid extraction; EO: essential oil obtained by hydrodistillation; HY: hydrolate obtained by hydrodistillation, tr: traces. Sensory description of volatile compounds mentioned in the literature.

**Table 3:** Mass composition of the selected molecules found in *T. mastichina* products, expressed in μg of compound per g of plant, in DW.

Compound	Mass (μg Compound·g Plant^−1^ (DW))					
	FR			DR		
	EX	EO	HY	EX	EO	HY
**α-Pinene**	15.9 ± 2.7 ^b^	8.6 ± 0.0 ^c^	-	6.5 ± 0.0 ^c^	65.0 ± 0.0 ^a^	-
**Camphor**	23.8 ± 11.6 ^a^	-	-	4.8 ± 0.7 ^b^	-	-
**Eucalyptol**	34.3 ± 7.4 ^b^	18.6 ± 1.1 ^c^	-	7.0 ± 0.1 ^d^	123.8 ± 9.7 ^a^	-
**γ-Terpinene**	87.2 ± 3.8 ^b^	64.1 ± 1.4 ^c^	25.1 ± 9.9 ^d^	111.5 ± 3.9 ^b^	609.4 ± 28.3 ^a^	2.6 ± 0.0 ^e^
***p*** **-Cymene**	49.5 ± 5.1 ^b^	29.0 ± 0.7 ^c^	14.8 ± 2.6 ^d^	55.6 ± 1.5 ^b^	337.0 ± 20.3 ^a^	1.7 ± 0.0 ^e^
***S*** **-Verbenone**	61.8 ± 21.1	-	-	-	-	-
**Thymol**	1511.3 ± 62.3 ^b^	404.4 ± 25.9 ^c^	2389.2 ± 39.2 ^a^	282.1 ± 7.5 ^c^	2565.5 ± 28.7 ^a^	-
**Total**	1783.8	524.8	2429.1	467.4	3700.7	4.3

Calibration curves: α-pinene (y = 1.02 × 10^10^x − 2.16 × 10^6^; *r*^2^ = 0.9987; LOD = 8.73 × 10^−4^ g∙L^−1^; LOQ = 2.64 × 10^−3^ g∙L^−1^), camphor (y = 9.03 × 10^9^x − 3.05 × 10^6^; *r*^2^ = 0.9974; LOD = 1.11 × 10^−3^ g∙L^−1^; LOQ = 3.37 × 10^−3^ g∙L^−1^), eucalyptol (y = 1.05 × 10^10^x − 4.86 × 10^6^; *r*^2^ = 0.9972; LOD = 1.29 × 10^−3^ g∙L^−1^; LOQ = 3.91 × 10^−3^ g∙L^−1^), γ-terpinene (y = 7.75 × 10^9^x − 4.89 × 10^6^; *r*^2^ = 0.9966; LOD = 1.44 × 10^−3^ g∙L^−1^; LOQ = 4.35 × 10^−3^ g∙L^−1^), *p*-cymene (y = 7.13 × 10^9^x − 2.66 × 10^6^; *r*^2^ = 0.9997; LOD = 4.05 × 10^−4^ g∙L^−1^; LOQ = 1.23 × 10^−3^ g∙L^−1^), *S*-verbenone (y = 7.60 × 10^9^x − 6.96 × 10^6^; *r*^2^ = 0.9946; LOD = 1.80 × 10^−3^ g∙L^−1^; LOQ = 5.47 × 10^−3^ g∙L^−1^), thymol (y = 2.45 × 10^9^x − 1.31 × 10^7^; *r*^2^ = 0.9977; LOD = 2.53 × 10^−3^ g∙L^−1^; LOQ = 7.66 × 10^−3^ g∙L^−1^). Averages with different letters in the same line indicate significant difference with α = 0.05. EX: extract obtained by carbon dioxide supercritical fluid extraction, EO: essential oil obtained by hydrodistillation, HY: hydrolate obtained by hydrodistillation, FR: fresh, DR: dry.

**Table 4:** Safe concentration ranges of the most promising *T. mastichina* products and commercial EO as defined by cytotoxic GI50 measurements and ODT values.

Test	FR (µg·mL^−1^)			DR (µg·mL^−1^)			Commercial EO Standard
	EX	EO	HY	EX	EO	HY	(µg·mL^−1^)
Vero cells (GI_50_)	235 ± 7 ^b^	122 ± 12 ^c^	>400 ^a^	271 ± 24 ^b^	65 ± 1 ^c^	>400 ^a^	83.5 ± 0.1 ^c^
ODT value	6.3 × 10^−4 a^	-	-	5.0 × 10^−3 a^	3.0 × 10^−4 a^	-	1.0 × 10^−4 a^

Averages with different letters in the same line indicate significant differences with α = 0.05. EX: extract obtained by carbon dioxide supercritical fluid extraction, EO: essential oil obtained by hydrodistillation, HY: hydrolate obtained by hydrodistillation.

## Data Availability

Not available.
